# Effectiveness of Nemolizumab in Improving Dialysis-Associated Pruritus Refractory to Difelikefalin: A Case Report

**DOI:** 10.7759/cureus.83367

**Published:** 2025-05-02

**Authors:** Yoshihito Mima, Masako Yamamoto, Ken Iozumi

**Affiliations:** 1 Department of Dermatology, Tokyo Metropolitan Police Hospital, Tokyo, JPN

**Keywords:** dialysis, difelikefalin, il-31, nemolizumab, peak pruritus numerical rating scale (pp-nrs)

## Abstract

Chronic kidney disease-associated pruritus (CKD-aP) is a common and distressing symptom among patients undergoing maintenance hemodialysis and is known to significantly impair quality of life. In contrast, prurigo nodularis (PN), characterized by intensely pruritic nodules, may develop as a result of persistent scratching due to chronic pruritus. While difelikefalin, a peripherally acting κ-opioid receptor (KOR) agonist, has been approved for the treatment of CKD-aP, alternative therapeutic options have not been sufficiently explored in patients who show an inadequate response. Herein, we report a case of a 74-year-old man on long-term dialysis who presented with severe CKD-aP (PP-NRS 9-10) and multiple pruriginous nodules. Although partial improvement was achieved with difelikefalin, moderate pruritus (PP-NRS 7) and residual nodules persisted. Nemolizumab, an interleukin-31 receptor α (IL-31Rα) antagonist approved for the treatment of PN, was subsequently introduced. After two doses, the patient experienced complete resolution of pruritus and marked flattening of nodular lesions. Elevated serum IL-31 levels have been reported in both PN and dialysis-related pruritus, suggesting a shared pathophysiological role of IL-31 in these conditions. The rapid and dramatic response to nemolizumab in this case supports the potential of IL-31 inhibition as a promising treatment strategy for patients with CKD-aP who are unresponsive to KOR agonists. This case represents the first report of sequential use of difelikefalin and nemolizumab for dialysis-associated pruritus and highlights the need for further studies to evaluate the efficacy of IL-31-targeted therapies in this setting.

## Introduction

Chronic kidney disease-associated pruritus (CKD-aP) is commonly observed in patients undergoing maintenance hemodialysis and is known to significantly impair quality of life due to complications such as sleep disturbances [1]. In addition, chronic kidney disease (CKD) is considered a risk factor for prurigo nodularis (PN), a chronic cutaneous inflammatory disease characterized by intensely pruritic nodules. Repetitive scratching due to persistent itch is thought to contribute to both the onset and chronicity of PN in these patients [2]. The pathophysiology of chronic pruritus in CKD involves multiple factors, including dysregulation of opioid signaling, peripheral neuropathy, activation of non-histaminergic pruritic pathways, and immune dysfunction triggered by the accumulation of uremic toxins [3]. Based on these mechanisms, difelikefalin, a peripherally acting κ-opioid receptor (KOR) agonist, was approved in 2021 for the treatment of moderate-to-severe pruritus in patients on dialysis [4]. In patients on dialysis, reduced KOR expression has been reported, and difelikefalin is believed to exert antipruritic effects by activating KORs expressed on peripheral neurons and immune cells [4]. In contrast, data on the efficacy of nemolizumab - an interleukin-31 receptor α (IL-31Rα) antagonist approved in 2024 as a novel treatment for PN - remain limited to clinical trial settings for dialysis-associated pruritus [5]. Here, we report a case of a patient with persistent dialysis-related pruritus despite initial treatment with difelikefalin. The patient presented with multiple pruriginous nodules on the extremities resulting from chronic scratching. The administration of nemolizumab led to a marked improvement in dialysis-associated pruritus, supporting its potential role as an alternative treatment in such cases.

## Case presentation

**Figure 1:**
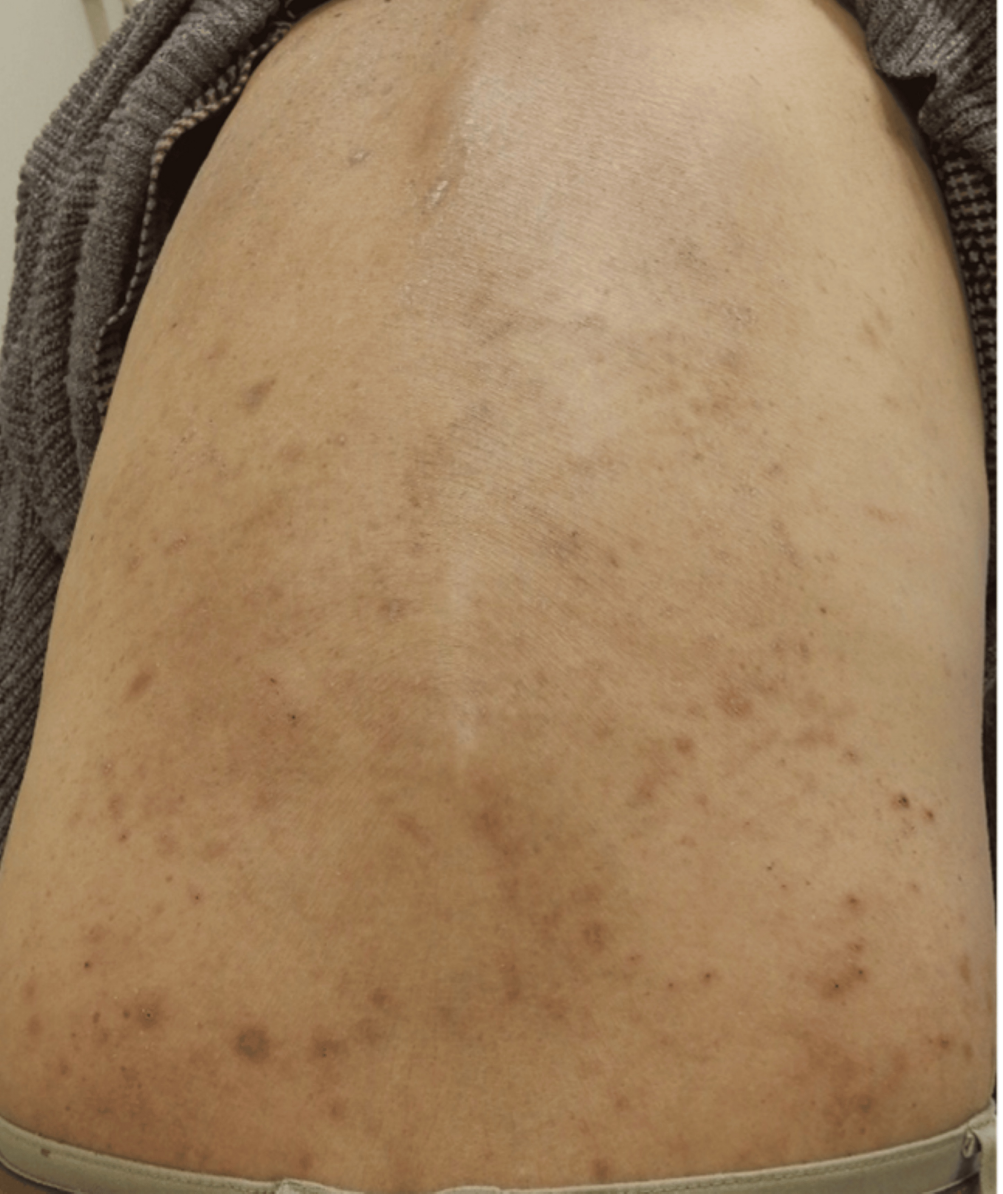
Difelikefalin treatment led to a marked improvement in scratch marks on the back, and individual pruriginous nodules showed a trend toward resolution. However, the nodules remained indurated with a palpable core and continued to be associated with persistent pruritus.

A 74-year-old man had been receiving maintenance hemodialysis for CKD for over five years. He was treated for pruritus and eczema with oral antihistamines and topical corticosteroids; however, his dialysis-associated pruritus remained poorly controlled. His peak pruritus numerical rating scale (PP-NRS) score ranged from 9 to 10, indicating severe and persistent itch. Difelikefalin acetate (35.0 μg per dose, administered three times weekly) was initiated, resulting in a modest improvement in the PP-NRS score to 7. Despite this, moderate pruritus persisted, prompting referral to our department for further therapeutic intervention. At the initial visit, physical examination revealed marked improvement in scratch marks and a trend toward flattening of many pruriginous nodules induced by chronic scratching. However, numerous nodules remained on the trunk and extremities (Figure 1).

Although some improvement in pruritus had been achieved, the patient continued to experience sleep disturbances due to itch and expressed a desire for intensified treatment. Targeting the residual pruritus and pruriginous nodules on the trunk and extremities, we initiated nemolizumab therapy (60 mg at baseline, followed by 30 mg monthly). Following the first dose, the PP-NRS score improved to 2, and after the second dose, pruritus completely resolved, demonstrating a remarkable therapeutic response. Given the complete remission of pruritus, nemolizumab was discontinued after two injections, with a plan to resume treatment if symptoms recur. The patient is currently being managed with maintenance therapy consisting primarily of emollients, with ongoing follow-up.

## Discussion

In this report, we described a case of dialysis-associated pruritus and PN secondary to chronic scratching, in which residual pruritus and pruriginous nodules were rapidly improved following treatment with nemolizumab after partial relief had been achieved with difelikefalin. The patient experienced significant relief from pruritus, resolution of sleep disturbances, and a marked improvement in quality of life, and expressed high satisfaction with nemolizumab treatment.

PN lesions are characterized by the infiltration of eosinophils, neutrophils, macrophages, mast cells, and T cells, with a predominance of Th2-type inflammation driven by CD4+ Th2 cells. In the dermis, increased expression of Th2 cytokines such as IL-4, IL-13, and IL-31 has been reported. Among these, IL-31 plays a pivotal role in the pathogenesis of PN by promoting fibrosis through fibroblast activation and enhancing pruritic signaling via neuroinflammatory mechanisms [6,7]. Multiple clinical trials have demonstrated that nemolizumab, an IL-31 receptor α (IL-31Rα) antagonist, effectively and rapidly alleviates pruritus and improves skin lesions in PN [6-8]. In the OLYMPIA trial evaluating the efficacy of nemolizumab for PN, 56.3% of patients in the nemolizumab group achieved a ≥4-point improvement in the PP-NRS score at week 16, compared with 20.9% in the placebo group (P < 0.001). Additionally, 37.7% of patients receiving nemolizumab achieved a Prurigo Nodularis Investigator’s Global Assessment (PN-IGA) score of 0 or 1 versus 11.0% in the placebo group (P < 0.001) [6]. Similarly, in patients undergoing dialysis, elevated serum IL-31 levels and upregulated IL-31 expression across the entire epidermis have been observed compared to healthy individuals [9,10]. Moreover, patients with dialysis-related pruritus exhibit significantly higher serum IL-31 levels than those without pruritus, suggesting a strong association between IL-31 and dialysis-associated pruritus [10]. In a clinical trial evaluating nemolizumab (0.5 mg/kg) for pruritus in dialysis patients, the visual analog scale (VAS) score for itch improved by 30.3 mm from baseline at week 1 and by 40.1 mm at week 4, demonstrating the efficacy of nemolizumab in dialysis-associated pruritus. Furthermore, the reduction in pruritus following nemolizumab treatment was greater in patients with higher baseline IL-31 levels compared to those with lower levels, further supporting the association between IL-31 and dialysis-related pruritus [5]. In the present case, the marked improvement in pruritus following nemolizumab administration supports the therapeutic potential of IL-31 inhibition for dialysis-associated cutaneous pruritus. Conversely, difelikefalin is believed to alleviate CKD-aP by correcting the imbalance between activation of μ-opioid receptors (MOR), which promote itch, and downregulation of κ-opioid receptors (KOR), which suppress itch. In addition, difelikefalin has been shown to suppress cutaneous IL-31 expression in patients with atopic dermatitis [11], suggesting that its antipruritic effects may involve a dual mechanism: KOR activation and IL-31 inhibition. In clinical trials with relatively short evaluation periods of 12 to 16 weeks, the overall incidence of adverse events (AEs) associated with nemolizumab ranged from 60% to 70%; this exceeded 80% in 24-week studies and surpassed 90% in long-term trials lasting 68 weeks [12]. However, most AEs were mild and manageable with intensified topical corticosteroid therapy, and treatment discontinuation due to AEs occurred in only 4-6% of cases, indicating a favorable safety profile [12]. In our own experience, no AEs have been observed during the six months following initiation of nemolizumab treatment.

To date, no reports have documented the combined or sequential use of difelikefalin and nemolizumab for dialysis-associated pruritus. In this case, difelikefalin provided partial symptom relief, yet moderate pruritus persisted, along with pruriginous nodules likely resulting from chronic scratching on the extremities. The introduction of nemolizumab led to a rapid and sustained resolution of pruritus. A limitation of our case is the lack of a definitive diagnosis by skin biopsy and the absence of regular follow-up with blood tests. Although nemolizumab is not currently approved for the treatment of CKD-aP, this case highlights its potential as an effective therapeutic option in patients who respond inadequately to difelikefalin.

## Conclusions

To date, there have been no reports on the combined use of difelikefalin and nemolizumab for dialysis-related pruritus. This case highlights that although not approved for CKD-aP, nemolizumab may be a promising option when difelikefalin is insufficient. Further research is needed.
